# A 5-year experience with an elective scholarly concentrations program

**DOI:** 10.3402/meo.v20.29278

**Published:** 2015-11-09

**Authors:** Paul George, Emily P. Green, Yoon S. Park, Philip A. Gruppuso

**Affiliations:** 1 Department of Family Medicine, The Warren Alpert Medical School of Brown University, Providence, RI, USA; 2 Section on Medical Education, The Warren Alpert Medical School of Brown University, Providence, RI, USA; 3 Student and Faculty Development, The Warren Alpert Medical School of Brown University, Providence, RI, USA; 4 Department of Medical Education, University of Illinois at Chicago, Chicago, IL, USA; 5 Department of Pediatrics, The Warren Alpert Medical School of Brown University, Providence, RI, USA

**Keywords:** undergraduate medical education, scholarly concentration, program evaluation

## Abstract

**Problem:**

Programs that encourage scholarly activities beyond the core curriculum and traditional biomedical research are now commonplace among US medical schools. Few studies have generated outcome data for these programs. The goal of the present study was to address this gap.

**Intervention:**

The Scholarly Concentration (SC) Program, established in 2006 at the Warren Alpert Medical School of Brown University, is a 4-year elective program that not only encourages students to pursue scholarly work that may include traditional biomedical research but also seeks to broaden students’ focus to include less traditional areas. We compared characteristics and academic performance of SC students and non-SC students for the graduating classes of 2010–2014.

**Context:**

Approximately one-third of our students opt to complete an SC during their 4-year undergraduate medical education. Because this program is additional to the regular MD curriculum, we sought to investigate whether SC students sustained the academic achievement of non-SC students while at the same time producing scholarly work as part of the program.

**Outcome:**

Over 5 years, 35% of students elected to enter the program and approximately 81% of these students completed the program. The parameters that were similar for both SC and non-SC students were age at matriculation, admission route, proportion of undergraduate science majors, and number of undergraduate science courses. Most academic indicators, including United States Medical Licensing Examinations scores, were similar for the two groups; however, SC students achieved more honors in the six core clerkships and were more likely to be inducted into the medical school's two honor societies. Residency specialties selected by graduates in the two groups were similar. SC students published an average of 1.3 peer-reviewed manuscripts per student, higher than the 0.8 manuscripts per non-SC student (*p*=0.013).

**Conclusions:**

An elective, interdisciplinary scholarly program with a focus beyond traditional biomedical research offers students the opportunity to expand the scope of their medical education without an untoward effect on academic performance or residency placement.

Programs that encourage medical students to engage in scholarly activities beyond the core curriculum are becoming more common among US medical schools (1). Many of these programs came into existence within the past 10 years, partially as a result of an effort carried out by a consortium of schools that first convened at the annual meeting of the Association of American Medical Colleges in 2007. Originally numbering 13, these schools were interested in sharing best practices regarding ‘Scholarly Concentration (SC) programs’, which can be defined as ‘educational programs that provide students with increased opportunities for in-depth inquiry’ (1–3). That group ultimately led to the formation of a ‘Scholarly Concentrations Collaborative’ that currently includes 52 medical schools, many of which have incorporated areas of study that do not appear in their core curricula into their SC programs. This expansion of this group is an indication of the widespread perceived value in the development of these programs.

Little information exists however regarding outcomes of these programs. Bierer and Chen's 2008 review (4) found 39 studies that addressed the impact of what these authors termed SC programs. Their definition included programs focused solely on student research and dual degree programs, such as MD-MPH and MD-PhD programs. These authors found that published studies were largely descriptive in nature or focused mainly on self-report measures and student satisfaction. In their conclusions, they advocated for increased rigor in evaluation designs to demonstrate the impact of SC programs.

Few studies addressing this gap in our knowledge have been forthcoming since Bierer and Chen's 2008 publication (4). A Medline search similar to the one these authors carried out, but excluding dual degree programs, yielded several papers. Most described novel medical student research programs (5) or offered perspectives on students’ attitudes toward medical student research (6, 7). Two studies, both of which addressed the optimization of research productivity, focused on required medical school research programs, thus precluding any comparative analyses regarding effectiveness of the programs (8, 9).

## SC Program at Alpert Medical School

The SC Program, established at the Warren Alpert Medical School (AMS) of Brown University in 2006, currently has 13 SCs, ranging from medical education to women's reproductive health to integrative medicine (Table 1). The AMS SC Program graduated its fifth cohort of participating students in May 2014. At the time of its inception, the SC Program at our institution was unusual with regard to its goals and desired outcomes. Perhaps, most unusual was that it aimed to promote scholarship beyond the scope of traditional biomedical research. At the time, AMS students benefited from an infrastructure that could support their participation in basic, clinical, and translational research. This infrastructure included the means to identify mentors and funding for summer assistantships. However, students interested in other scholarly pursuits, particularly those in the humanities and social sciences, lacked such an infrastructure. To provide them with the resources and structure to successfully undertake scholarship in diverse areas related to medicine resulted in the establishment of the SC Program and the commitment of resources to administer it.

**Table 1:** Examples of scholarly concentration educational components and final projects

Scholarly concentration	Didactic/experiential components	Representative projects
Advocacy and activism	Discussion sessions on societal and economic consequences of disparities in the provision of care	Putting a face to Providence's homeless veteran population Socioeconomic disparities in cancer care: a broad-based analysis
Aging	Lecture series on geriatric and psychiatric medicine	Changes in IL-6 and CRP levels in post-operative orthopedic patients Eye injuries in the elderly
Caring for underserved populations	Lecture series on health disparities	Qualitative study on why individuals who live in poorer communities smoke
Contemplative studies	Attendance at a contemplative retreat	Yoga for PTSD Hypnotherapy versus gabapentin in the treatment of hot flashes
Disaster medicine	Lecture series on how systems respond to healthcare needs during and after a disaster	Injury and illness patterns in workers during the 9/11 rescue and recovery operation at the World Trade Center Variations in the seasonal risks of an aerosolized bioterror attack
Global health	Global health seminar series	Public Health Education and Training Program for Youth in Rural Haiti Nyaya Health: a public-private partnership to develop healthcare capacity in rural Nepal
Health policy	Lecture series on topics such as Medicare, Medicaid, and the Affordable Care Act	Scholarly paper on the effect of the Sunshine Act on physicians
Integrative medicine	Alternative Medicine seminar series	Participation in a acupuncture clinic at a community hospital in an underserved community
Medical education	Medical education seminar series	From student to mentor: making a difference in the lives of LGBT teens Development of a student-based teaching academy
Medical humanities and ethics	Medical humanities seminar course	Paired narratives of shared experiences around chronic low back pain: classic mismatch between patients and their doctors Comfort feeding only: a proposal to bring clarity to decision making regarding difficulty with eating for persons with advanced dementia
Medical informatics	Topics in Translational Research seminar series	Use of percutaneous, image-guided therapies in cancer treatment
Physician as a communicator	Physician as a Communicator pre-clinical elective course	Beyond pain and meds: stories of chronic pain patients on long-term opioids Body, text, and formaldehyde
Women's reproductive health	Women's Reproductive Health seminar series	Physical activity levels in women with pelvic floor disorders Labor and delivery nurses’ attitudes, beliefs, and knowledge of emergency contraception

CRP, C-reactive protein; LGBT, lesbian, gay, bisexual, and transgender.

The decision to establish the SC Program was based on several critical factors. One was our institution's broadly defined mission, which includes but is not particularly focused on the training of physician scientists. Another was the principle that students would benefit educationally from undertaking a research project only if they understood the purpose of doing so and were self-directed. The third principal was that requiring scholarship beyond the already intense, core requirements for the MD at our institution may potentially result in lower quality scholarship. It was this last factor that led to the decision that the SC Program be elective in nature.

The program is interdisciplinary in nature. Concentration areas are not meant to prepare students for specific specialties or to directly enhance residency applications to those specialties by demonstrating early specialty commitment. Instead, students are encouraged to identify or develop projects that cross traditional disciplinary boundaries, or that involve multiple areas in the biomedical sciences and humanities. To this end, the SC Program offers students a broad array of concentration areas from which to choose (Table 1). In developing the program, two frameworks were used. The first is Lave and Wenger's community of practice theory (10) in which students participate in a community (in this case, one of the SCs), create a shared understanding of what led them to this community (their mutual interest in a topic related to but not often taught in medical school), and produce a scholarly project (which becomes a resource for others). The second framework we used was self-determination theory (11), in which students have a sense of relatedness to faculty within their SC, have a sense of autonomy in developing their project, and a sense of competence once they complete the SC in a topic not typically taught in medical school.

Students apply to the SC Program in the spring of their first year at medical school. Applications consist of a research proposal for 8–10 weeks of full-time work in their area of interest to be completed during the summer months. This summer immersion experience is a required component of the SC Program. The proposal must also contain a plan for how that summer work will extend or become a longitudinal project that will last for the duration of a student's medical education. Students applying to the SC Program are eligible to receive a stipend from AMS and traditionally have been highly successful in receiving funding. During years 2 through 4 of medical school, students continue their project work while participating in other required activities. Students submit a scholarly product in the fourth year, the nature of which is flexible and intended to best suit the student's academic focus.

In order to better determine the impact of SC programs on medical students, more information is needed about the relationship between participation in these programs and academic performance, indicators of academic excellence, career choice, and scholarly productivity. While the program has become an established component of the educational landscape at our institution, and one that has proven popular with applicants, students, and participating faculty, we considered it important for our institution (and for us as leaders in the SC Program) to assess the effectiveness of the program in promoting desired medical education outcomes. To that end, we hypothesized students in the SC Program would produce more scholarly research than non-concentrators, and achieve the same or better educational outcomes as non-concentrators, despite participation in this intense, non-required program.

## Methods

The study was approved by our university's institutional review board. The study cohorts included all medical students, except for those in our MD/PhD program, who graduated between 2010 and 2014. We extracted data from students’ academic records and the records of the SC Program. These data included demographics of participants and non-participants in the SC Program and, for the former, participation in specific concentrations. We also included age at matriculation, gender, and representation of traditionally underrepresented groups. Finally, we examined students’ admission routes to medical school: the traditional premedical route [in which students take typical premedical courses and the medical college admissions test (MCAT)]; participation in our 8-year combined baccalaureate/MD program; the Program in Liberal Medical Education (PLME) (12), which contributes about 40% of matriculants (in which the MCAT is not required); or routes other than these two such as a post-baccalaureate route (in which students take premedical courses after undergraduate education and may or may not take the MCAT). We also captured data on premedical major (science vs. non-science) and undergraduate courses (science vs. non-science).

Academic metrics included performance in the United States Medical Licensing Examinations (USMLE) Step 1 and USMLE Step 2 Clinical Knowledge (CK) examinations during the first 2 years of medical school, number of honors grades in core clerkships (this is based on end of clerkship exam scores (shelf exams produced by the National Board of Medical Examiners), objective structured clinical examination scores, and direct observation; approximately 30% of students receive honors in each clerkship), admission to the Alpha Omega Alpha (AOA) Medical Honor Society, and admission to the Gold Humanism Honor Society (GHHS). We also compared the residency specialty choices of SC Program participants and non-participants.

We defined scholarly productivity as the number of publications in peer-reviewed journals and the impact factors of those journals. To gather these data, we used PubMed, querying students’ first and last names, and our institution's name as search terms. For results that were ambiguous, we accessed the journal article to verify that the author was indeed a medical student at our institution. We included articles published till January 1, 2014, by members of classes of 2010 through 2013. Publication data for the Class of 2014 were thought to be too incomplete at the time of analysis for inclusion in this portion of our study.

We analyzed the data using GraphPad Prism (GraphPad Software, Inc., La Jolla, CA) and SPSS Version 22 (SPSS, Inc., 2012, Chicago, IL, www.spss.com). We first analyzed descriptive statistics and then used unadjusted tests of means and proportions (independent *t*-tests and chi-squared tests) to compare variables associated with students who participated in the SC Program with those who did not. We then performed logistic regression analysis for dichotomous outcome measures (those measures which had only two possible results such as induction into the AOA or GHHS). For continuous outcome measures, linear and logistic regressions controlled for age, underrepresented minority status, and gender, to better isolate the effect of the SCs on student performance (13).

## Results

**Fig. 1:**
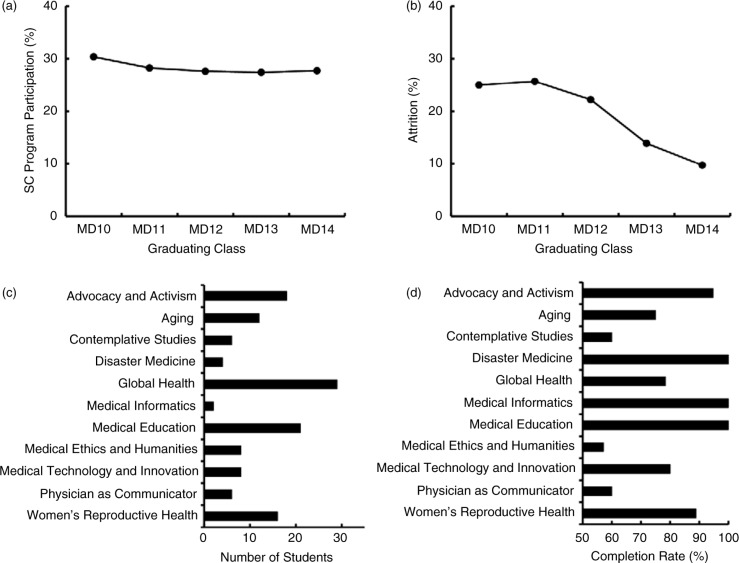
Participation in AMS’ elective SC Program. Data from the first five graduating classes to participate in the program were analyzed for percent of students choosing to participate in the program (a), attrition prior to graduation, defined as the percent of students who started the program who did not complete it (b), distribution of students among specific concentration areas (c), and completion rate for the various concentrations (d).

For the MD classes of 2010–2014, students could choose among 13 available SCs. Based on their diverse areas of focus, individual concentrations utilized various curricular methodologies and yielded a wide variety of final projects (Table 1). Among the 460 students in all five graduating classes, 161 (35.0%) applied for and were accepted to an SC. Of these, 130 (28.3%) submitted a final project and were acknowledged at graduation as having completed a SC. Completion of the program has been consistent from year to year (Fig. 1a). However, the rate of attrition (Fig. 1b) has shown a decreasing trend over the past 3 years. Distribution among specific concentrations (Fig. 1c) has varied considerably from year to year, as has the percentage of students within specific concentrations who began the program but did not complete it (Fig. 1d).

**Fig. 2:**
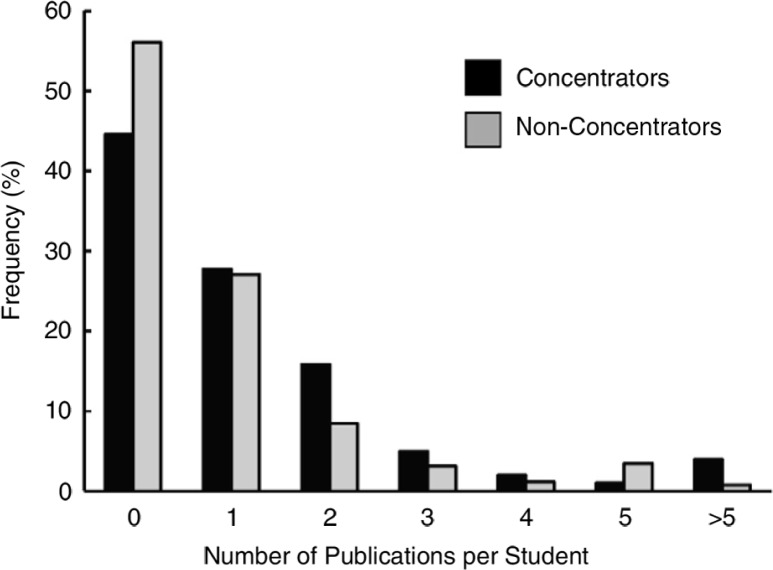
Distribution of the number of publications per student. Data are shown for concentrators and non-concentrators.

The comparative demographics of SC students and non-SC students (Table 2) revealed few differences between the two groups. Students’ age at matriculation, route of admission to AMS, proportion of undergraduate science majors, and number of undergraduate science courses were all similar for SC students and non-SC students. There was a slight overrepresentation of women among SC students. The only other difference identified within the demographic data was a smaller than expected proportion of SC students, relative to non-SC students, who came from traditionally underrepresented groups.

**Table 2:** Demographic data

	Concentrators (*N*=130)	Non-concentrators (*N*=330)	Effect size	Statistical analysis
Matriculation age (years)	22.9 (2.6)^a^	22.6 (2.4)	0.122	*p*=0.239
Gender (percentage female)	60.0%	51.1%	0.179	*p*=0.049
Admission route (percent of total)				
Standard premed	40.0%	31.7%	0.173	*p*=0.211
PLME	50.8%	55.9%	0.102	
Other	9.2%	12.7%	0.112	
Percentage underrepresented minority	9.2%	21.1%	0.338	*p*=0.002
Percentage undergraduate science majors	48.5%	53.0%	0.090	*p*=0.376
Number of undergraduate science courses	12.5 (6.6)	11.6 (5.9)	0.147	*p*=0.143

PLME, Program in Liberal Medical Education.

a Comparison of demographics between concentrators and non-concentrators including age, admission route, number of underrepresented minority students, and undergraduate major.

Numbers in parentheses represent one standard deviation. For effect sizes, Cohen's *d* was calculated for differences in means; Cohen's *h* was calculated for differences in proportions.

We examined a number of academic performance measures (Table 3). SC students and non-SC students did not differ with regard to mean USMLE Step 1 score, the aggregate examination average for years 1 and 2, the mean clerkship subject examination (‘shelf exam’) score, or the mean USMLE Step 2 CK score. However, SC students showed a significantly higher rate of achieving a grade of honors in the six core clerkships. The significance of this difference persisted after controlling for age, gender, and underrepresented minority status in a linear regression model.

**Table 3:** Academic performance data^a^

	Concentrators (*N*=130)	Non-concentrators (*N*=330)	Effect size	Statistical analysis
Step 1 three-digit score	227.2 (19.9)	225.1 (22.6)	0.096	*p*=0.387
Average year 1–2 examination score	85.5 (5.9)	85.2 (5.8)	0.051	*p*=0.674
Step 2 CK score	240.5 (17.9)	237.2 (21.8)	0.159	*p*=0.198
Average clerkship examination score	78.6 (6.6)	77.3 (7.4)	0.181	*p*=0.315
Number of clerkship honors per student	2.8 (1.8)	2.0 (1.8)	0.444	*p<*0.001
Percent inducted into AOA	24.5	14.1	0.266	*p*=0.022
Percent inducted into GHHS	25.3	12.1	0.344	*p*<0.001

AOA, Alpha Omega Alpha; GHHS, Gold Humanism Honor Society.

a Comparison of outcome factors between concentrators and non-concentrators, including Step 1 score, average Year 1 and Year 2 score, Step 2 Clinical Knowledge (CK) score, average clerkship examination score; average number of clerkship honors and number of students inducted into Alpha Omega Alpha and the Gold Humanism Society.

Numbers in parentheses represent one standard deviation. For effect sizes, Cohen's *d* was calculated for differences in means; Cohen's *h* was calculated for differences in proportions.

Students participating in the SC Program were more often inducted into the AOA (Table 3). They were also more likely to be admitted to the GHHS (Table 3). The significance of these differences persisted after controlling for age, gender, and underrepresented minority status in a logistic regression model.

The two groups were similar with regard to specialty areas chosen in the residency match. There was no statistically significant difference between SC students and non-SC students entering primary care residencies (81 of 125, 64.8%, for SC students vs. 157 of 311, 50.5%, for non-SC students, *p*=0.15). Likewise, there was no statistical difference in students entering surgical specialties (7 of 125, 5.6%, for SC students vs. 28 of 311, 9.0%, for non-SC students, *p*=0.27). An indirect measure of the competitiveness of the specialties chosen by the two groups, the mean Step 1 score for US medical students matching in specific specialties, was not different while comparing the two groups: 223.1 (7.4) for SC students versus 224.8 (15.3) for non-SC students (*p*=0.25). The interpretation of these data led to the premise that completion of the SC Program was not associated with differences in career choice or residency placement relative to the group of non-SC students.

Scholarly productivity for the two groups was measured as the number of peer-reviewed publications per student and the impact factors of the journals in which the students’ work was published. SC students published an average of 1.29 papers per student, a slightly higher figure than the 0.83 papers published per non-SC student. The frequency of publications by SC students and non-SC students is shown in Fig. 2. This difference was statistically significant (*p*=0.013 controlling for age, gender, and underrepresented minority status). The mean impact factor of journals in which SC students and non-SC students published their work was similar: 2.91 and 3.50, respectively; non-significant by Mann–Whitney U test.

As referenced above, 31 of the 161 students (19.3%) who initially applied to and were accepted into the SC Program did not complete the program. The members of this group did not differ from non-SC students with regard to age at matriculation, gender, or admission route. They were less likely to be science majors as undergraduate students (12 of 31, 38.7%). They did not differ from either group in any of the quantitative indicators of academic performance. However, only 2 of 26 students in this group (7.1%) were inducted into the AOA. They did not differ from the other two groups with regard to residency placement. Their rate of peer-reviewed publication was lower than the other two groups at 0.52 publications per student.

## Discussion

Given the elective nature of the SC Program at AMS, one question was whether or not program participation and, by extension, sustainability of the program would be maintained. We found that student participation has been remarkably consistent. We speculate that as the program has become better known and understood by our students, their decision to apply to the program may be better informed. In addition, students often discuss their SC work during residency interviews and receive letters from their SC mentors, which also adds value to the program, especially in light of residency positions being available at a premium.

From a theoretical standpoint, we speculate that one reason why the program is successful is the fulfillment of the theories described in the introduction. The discussions and didactic sessions (often led by interdisciplinary faculty members, such as physicians, lawyers, ethicists, and others), and the pairing of medical students with faculty around shared interests, are at the heart of each SC area. These activities facilitate the development of a community of practice for students. The relationships formed with like-minded faculty and peers serve as part of participating students’ professional development. From a self-determination theoretical standpoint, students function autonomously in selecting their project (with coaching from their mentors) and achieve competence in a domain again not typically covered in undergraduate medical education.

The variation in attrition rate among concentration areas has also informed our approach to program design and coordination. According to information gathered through exit questionnaires and personal conversations with students, highly demanding didactic requirements both within the SC Program and in the regular curriculum have, in some cases, contributed to attrition as has inconsistent mentoring. Inconsistent mentoring especially has led to greater attrition from some of the SCs. In response, we evaluate the SCs regularly and provide feedback (and occasionally replace) mentors who may not be as effective.

Participation in the SC Program has been independent of admission route. This was somewhat unexpected given that about half of our student body comes to the school via a combined baccalaureate/MD program. The PLME encourages undergraduate students to explore areas outside the traditional premedical disciplines. We anticipated that students who entered AMS through such a program would be more likely to elect a program that intends to promote a ‘liberal education’ experience in medical school. However, because AMS has the PLME program, we do not have data for MCAT scores or undergraduate grade point averages (neither are required for admittance to AMS) to compare scholarly concentrators from non-concentrators at baseline. This is a limitation of our study. In addition, our admission process does not objectively measure a student's propensity to be self-directed. This raises the possibility of selection bias in our study, as students who enroll in the SC may be more self-directed than those who do not.

The one demographic characteristic that differed between SC students and non-SC students was the lower percent of students from traditionally underrepresented groups. The results of our analysis do not allow us to account for this observation. It may be that the population of students from traditionally underrepresented groups coincides with a higher proportion of students from less advantaged educational backgrounds. Given the demanding nature of medical education, it may be that some students opt out of participating in the SC Program in order to focus more of their efforts on the core, required curriculum. This difference in participation by students from underrepresented groups did not account for any of the observed differences between the SC student and non-SC student groups. Regardless, future efforts must be made to rectify this disparity.

SC students and non-SC students had similar outcomes with regard to academic performance. However, there was a highly significant difference in the number of clerkship honors grades, nearly three per SC students versus just over two per non-SC students. We must point out though, because this is not a randomized control trial (but only a quasi-experimental design), our results only demonstrate an association between participation in the SCs and academic success. We do, however, believe these results will be useful to other institutions with similar programs (or implementing them). Further randomized studies, if possible, would help in determining whether participation in the SCs truly do lead to greater academic success of participants although these types of studies are often difficult to perform in educational settings.

SC students also outperformed non-SC students in another qualitative indicator of academic performance, selection to the AOA honor society. This may be a reflection of the selection criteria for AOA at our institution, which include not only quantifiable academic performance measures, such as grades and licensing exam scores, but also accomplishments in extracurricular areas, which may include participation in an SC. The same contributing factors may account for the higher numbers of concentrators who were selected by their peers and faculty for induction into the GHHS.

SC students chose to pursue careers in primary care disciplines in approximately the same numbers as did non-SC students. Our data indicate that neither the decision on the part of a student to participate in the SC Program nor the impact of the SC Program on a student's educational experience is related to postgraduate career choice.

Participation in the SC Program was associated with increased scholarly productivity measured as the number of peer-reviewed publications per student, although there was no difference in the mean (and relatively high) impact factor between the two groups. Given that the program was intended to provide students with mentorship, time, and funding to pursue their work, the results of our analysis are consistent with the program having achieved this goal. Our data are also consistent with the conclusion that the SC Program offers students the opportunity to undertake interdisciplinary, and often creative, scholarly work without an untoward effect on academic performance or residency placement.

The SC Program offers some benefits to AMS and its students that are not reflected in our analysis. The interdisciplinary focus of the program contributes to the culture at AMS and to the recruitment of a student body with broad and diverse interests. The scholarly work undertaken by students who elect to participate in the SC Program reflects the diversity of the fields of study within the discipline of medicine. That said, further studies are warranted to examine the performance of our graduates as residents and attending physicians, their future scholarship, and the institutional impact of SC programs across the increasing number of institutions that offer them.
